# Certain Environmental Conditions Maximize Ammonium Accumulation and Minimize Nitrogen Loss During Nitrate Reduction Process by *Pseudomonas putida* Y-9

**DOI:** 10.3389/fmicb.2021.764241

**Published:** 2021-12-13

**Authors:** Xuejiao Huang, Wenzhou Tie, Deti Xie, Daihua Jiang, Zhenlun Li

**Affiliations:** ^1^Key Laboratory of (Guang Xi) Agricultural Environment and Products Safety, College of Agronomy, Guangxi University, Nanning, China; ^2^Chongqing Key Laboratory of Soil Multiscale Interfacial Process, Southwest University, Chongqing, China

**Keywords:** *Pseudomonas putida* Y-9, nitrate reduction, carbon source, C/N ratios, pH, dissolved oxygen, *nirBD* expression

## Abstract

Realizing the smallest nitrogen loss is a challenge in the nitrate reduction process. Dissimilatory nitrate reduction to ammonium (DNRA) and nitrate assimilation play crucial roles in nitrogen retention. In this study, the effects of the carbon source, C/N ratio, pH, and dissolved oxygen on the multiple nitrate reduction pathways conducted by *Pseudomonas putida* Y-9 are explored. Strain Y-9 efficiently removed nitrate (up to 89.79%) with glucose as the sole carbon source, and the nitrogen loss in this system was 15.43%. The total nitrogen decrease and ammonium accumulation at a C/N ratio of 9 were lower than that at 12 and higher than that at 15, respectively (*P* < 0.05). Besides, neutral and alkaline conditions (pH 7–9) favored nitrate reduction. Largest nitrate removal (81.78%) and minimum nitrogen loss (10.63%) were observed at pH 7. The nitrate removal and ammonium production efficiencies of strain Y-9 increased due to an increased shaking speed. The expression patterns of *nirBD* (the gene that controls nitrate assimilation and DNRA) in strain Y-9 were similar to ammonium patterns of the tested incubation conditions. In summary, the following conditions facilitated nitrate assimilation and DNRA by strain Y-9, while reducing the denitrification: glucose as the carbon source, a C/N ratio of 9, a pH of 7, and a shaking speed of 150 rpm. Under these conditions, nitrate removal was substantial, and nitrogen loss from the system was minimal.

## Highlights

The roles of DNRA and assimilatory reduction during NO_3_^–^ removal and nitrogen conservation in soils have been insufficiently examined. Moreover, the effects of environmental factors on the NO_3_^–^ reduction process when the three NO_3_^–^ reduction pathways (denitrification, DNRA, and assimilation) coexist remain unclear. In this study, the effect of the carbon source, C/N ratio, pH, and dissolved oxygen on ammonium accumulation and the expression of *nirBD* in strain Y-9 are explored during the nitrate reduction processes. The following conditions facilitated nitrate assimilation and DNRA by strain Y-9 while simultaneously reducing denitrification: glucose as the carbon source, a C/N ratio of 9, a pH of 7, and a shaking speed of 150 rpm. Under these conditions, nitrate removal was substantial, and nitrogen loss from the system was minimal. These findings provide theoretical support for technical studies of nitrate removal and nitrogen retention in soils.

## Introduction

Large amounts of industrial fertilizers are often applied to crops to increase crop yields. This leads to the considerable accumulation of nitrate (NO_3_^–^) in the soil (Kraft et al., 2014). NO_3_^–^, a mobile anion, is prone to loss by denitrification or runoff into surface waters, and this not only decreases the efficiency of nitrogen fertilizers but also has various passive environmental impacts including water eutrophication and greenhouse gas (nitrous oxide, N_2_O) emissions (Beeckman et al., 2018; Li et al., 2018; Sánchez and Minamisawa, 2019; Xia et al., 2020). The ammonium (NH_4_^+^), produced by the dissimilatory reduction of NO_3_^–^ to NH_4_^+^ (DNRA) via microorganismal respiration, can be adsorbed by soil colloids and then utilized by crops (Song et al., 2014; Zhang et al., 2015; Pandey et al., 2019). Similarly, the microbial assimilatory reduction of NO_3_^–^ can reduce NO_3_^–^ to NH_4_^+^ via NO_2_^–^ catalyzed by the relative reductase. Then, the NH_4_^+^ is incorporated into biomolecules and used by the bacterium. After death, the microorganisms release the NH_4_^+^ via mineralization for plant use (Shao et al., 2011; Wang et al., 2020). It is clear that DNRA and NO_3_^–^ assimilation ease the accumulation of NO_3_^–^ in agricultural soils and improve the efficiency of nitrogen fertilizers. These processes reduce the risk of NO_3_^–^ loss and mitigate the adverse effects of nitrogen fertilizer use. Several recent studies have investigated the role of DNRA in soil nitrogen conservation in farmlands (Shan et al., 2016; Friedl et al., 2018). Yet, the important role of assimilatory NO_3_^–^ reduction in NO_3_^–^ removal and nitrogen conservation in soils has been comparatively neglected.

Several environmental factors, including the C/N ratio, oxygen concentration, carbon source, affect enzyme activity in microorganisms by controlling the expression of relevant genes (e.g., *amoA*, *hao*, *narG*, and *nirK*) and thus impacting nitrogen cycles (Szukics et al., 2010; Ke et al., 2013; Caranto and Lancaster, 2017; Yu et al., 2019). Thus, we speculated that soil NO_3_^–^ removal could be maximized and soil nitrogen loss could be minimized by adjusting certain external environmental factors to enhance NO_3_^–^ assimilation and DNRA while decreasing denitrification. Typically, higher C/N ratios favor DNRA over denitrification (Kraft et al., 2014; Yoon et al., 2015; Van den Berg et al., 2016; Putz et al., 2018). Some studies have reported that glucose addition improved the NO_3_^–^ assimilation capacity of the soil (Recous et al., 1990; Romero et al., 2015). However, the effects of environmental factors on the NO_3_^–^ reduction process when the three NO_3_^–^ reduction pathways coexist are unclear.

*Pseudomonas putida* Y-9 performs NO_3_^–^ assimilation, DNRA, and denitrification under aerobic conditions simultaneously. The gene *nirBD* has been shown to control the assimilation and DNRA process (Huang et al., 2020). In this study, we investigate the effects of the carbon source, C/N ratio, pH, and dissolved oxygen (DO) on the accumulation of ammonium in the medium and the expression of *nirBD* in strain Y-9 during the nitrate reduction process. This study focuses on adjusting the environmental factor parameters to enhance the DNRA and NO_3_^–^ assimilation of strain Y-9. The results will provide theoretical support for technical research on NO_3_^–^ removal and nitrogen retention in soil.

## Materials and Methods

### Microorganisms and the Culture Media

*P. putida* Y-9 (Genbank No. KP410740), which performs NO_3_^–^ assimilation, denitrification, and DNRA under aerobic conditions simultaneously (Huang et al., 2020), was used in this study.

A denitrification medium (DM) was used to assess the nitrate reduction abilities of strain Y-9. The DM (per liter, pH = 7.2) contained 7.0 g K_2_HPO_4_, 3.0 g KH_2_PO_4_, 5.13 g CH_3_COONa, 0.10 g MgSO_4_ ⋅ 7H_2_O, 0.72 g KNO_3_, and 0.05 g FeSO_4_ ⋅ 7H_2_O. Luria-Bertani (LB) medium used for bacterial enrichment contained 10 g NaCl, 10 g tryptone, and 5 g yeast extract per liter (per liter, pH 7.0–7.2). All of the mediums were autoclaved for 30 min at 121°C.

### Effects of the Different Factors on Nitrate Reduction

The preserved strain Y-9 bacteria were activated in the LB medium at 150 rpm and 15°C for 36 h. Cells in the logarithmic growth phase were inoculated into a DM medium to assess the effects of the carbon source, C/N ratio, pH, and DO on Y-9–driven NO_3_^–^ reduction (Li et al., 2019; Yan et al., 2021).

In the carbon source experiments, one of the three carbon sources (sodium acetate, glucose, or sodium citrate) was added to 100 mL of DM medium. The C/N ratio, pH, and shaking speed were kept constant at 15, 7, and 150 rpm, respectively. In the C/N ratio experiments, 100 mL aliquots of the DM medium were amended with glucose to yield C/N ratios of 3, 6, 9, 12, or 15. The pH and shaking speed were kept constant at 7 and 150 rpm, respectively. In the pH experiments, the initial pH was adjusted using NaOH and HCl to 4, 6, 7, 8, or 9. The carbon source was glucose, and the C/N ratio and shaking speed were held constant at 9 and 150 rpm, respectively. To determine the effects of DO on NO_3_^–^ reduction, the shaking speed was set to 0, 50, 100, 150, or 180 rpm according to previous studies (Ren et al., 2014; Lei et al., 2019; Chen et al., 2021; Yan et al., 2021). The carbon source was glucose, and the C/N ratio and pH were kept constant at 9 and 7, respectively. The cultures were incubated at 15°C for 4 d. All of the above experiments were performed in triplicate. Samples were taken every day from each system. The optical density at 600 nm (OD_600_), NH_4_^+^, NO_3_^–^, and total nitrogen (TN) were measured for each sample.

### Kinetic Analysis of Nitrate Degradation

The modified Compertz model was used to describe the kinetics analysis of nitrate degradation by strain Y-9 (Chen et al., 2016). The kinetic equation was $\text{y}={\text{y}}_{0}\,\left(1-\exp\,\left(-\exp\,\left(\frac{\text{e}\,R_{m}}{{\text{y}}_{0}}\,\left({\text{t}}_{0}-t\right)\,1\right)\right)\right)$, where y is the NO_3_^–^ concentration at different incubation times (mg/L); y_0_ is the initial concentration of NO_3_^–^ (mg/L), *R*_m_ is the maximum conversion rate (mg/L/h), t_0_ is the lag time (h), t is the reaction time (h), and e is the mathematical constant.

### Expression of *nirBD* in Strain Y-9

Total RNA was extracted from strain Y-9 after 4 d of incubation under various conditions using a Trizol extraction kit (Invitrogen, United States), following the manufacturer’s instructions. The specific primers B1/B2 (F: CGCAACCATCTGCTCGTGT; R: CTGGCGGGTGTAGGAAAAGT) were designed based on the *nirBD* gene sequence (GenBank, MK561362). These primers were used to amplify the *nirBD* gene from the isolates. The 16S rRNA gene was used as an internal standard, as structural rRNA is present in cells at reasonably constant levels under normal growth conditions (Edwards and Saunders, 2010). The 16S rRNA gene was amplified using the forward primer GAACGCTAATACCGCATACGTCC and the reverse primer ATCATCCTCTCAGACCAGTTAC. The total RNA was reverse-transcribed using the RevertAid first-strand cDNA synthesis kit following the manufacturer’s instructions. Real-time quantitative PCRs were performed using the SYBR^®^ Premix Ex Taq™ II. Each real-time PCR was performed in triplicate. The PCR cycling conditions were as follows: initial denaturation at 95°C for 30 s; 38 cycles of 95°C for 15 s, 60°C for 30 s, and 72°C for 30 s; 1 cycle of 95°C for 15 s; and, finally, stepwise temperature increases from 55°C to 95°C to generate the melting curve. Standard curves were established using a dilution series of pMD19-T vectors containing the target gene.

### Analytical Methods

The OD_600_ was determined based on the absorbance at 600 nm, which was measured using a spectrophotometer. The contents of the different forms of nitrogen were determined as described by Huang et al. (2019). TN was measured in the suspension. The concentration of NH_4_^+^, NO_3_^–^, and NO_2_^–^ was measured in the supernatant, which was obtained by centrifuging each sample at 8,000 rpm for 5 min. Three replicates were analyzed per sample, and the results are presented as means ± the standard deviation of the mean (SD). The TN and NO_3_^–^ removal efficiencies were calculated as follows:*R*_*V*_ = (T_1_−T_2_)/T_1_×100%, where *R*_v_ is the removal efficiency of TN or NO_3_^–^ (%), and T_1_ and T_2_ are the initial and final concentrations of TN or NO_3_^–^ in the system, respectively.

### Statistical Analyses

One-way analyses of variance (ANOVAs), followed by Duncan’s Multiple Range Tests were performed using SPSS 22, and the differences among means were considered statistically significant at *P* < 0.05. Graphs were drawn using Origin 8.6 and GraphPad Prism 6.

## Results and Discussion

### Effects of the Carbon Source on Nitrate Reduction

**FIGURE 1:**
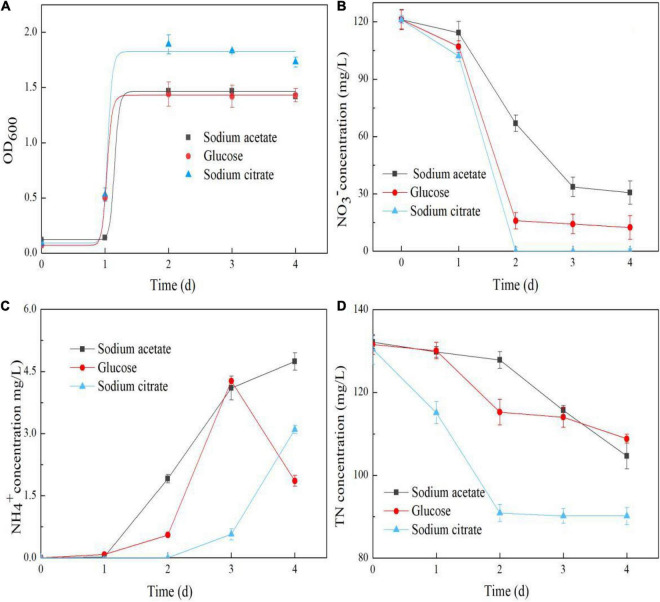
Effects of carbon type on the OD_600_ **(A)**, NO_3_^–^ concentration **(B)**, NH_4_^+^ concentration **(C)**, and TN concentration **(D)** in the *Pseudomonas putida* Y-9 culture.

A carbon source is typically essential for the growth of heterotrophic microorganisms, and it acts as an electron donor for nitrogen cycling (Sun et al., 2016). In this study, strain Y-9 grew vigorously and reached the stationary cell growth phase after 2 d when sodium acetate, glucose, or sodium citrate was used as the sole carbon source (Figure 1A). Moreover, sodium acetate, glucose, and sodium citrate were suitable carbon sources for NO_3_^–^ removal, with removal efficiencies of 74.75, 89.79, and 100%, respectively, at 4 d (Figure 1B). These results were consistent with those of Guo et al. (2016), who reported that sodium acetate, glucose, and sodium citrate enhanced the NO_3_^–^ removal capacity of *Enterobacter cloacae* strain HNR. Furthermore, the nitrate degradation rate followed the modified Compertz model (*R*^2^> 0.90), and the maximum NO_3_^–^ conversion rates were 1.60, 4.92, and 44.35 mg/L/h in media containing sodium acetate, glucose, and sodium citrate, respectively (Table 1).

**TABLE 1:** Kinetic parameters and final removal efficiency for the degradation of nitrate by strain Y-9 under different environmental conditions.

	Environmental factor	*R*_m_ (mg/L/h)	t_0_(h)	*R* ^2^	The last nitrate removal efficiency (%)
Carbon sources	Sodium acetate	1.6	20.97	0.94	74.14
	Glucose	4.92	23.99	0.97	89.79
	Sodium citrate	44.35	23.82	1	100
C/N	3	0.44	9.35	0.69	30.46
	6	0.58	−21.1	0.64	54
	9	1.91	5.17	0.9	81.78
	12	5.44	16.99	0.99	100
	15	3	11.68	0.97	89.79
pH	4	−1.63	−3177.13	−3.21	4.99
	6	0.8	−7.7	0.74	58.27
	7	2.05	4.87	0.90	81.78
	8	1.91	10.99	0.87	80.57
	9	1.5	6.04	0.87	79.14
Shaking speed	0	1.33	38.7	0.97	69.9
	50	1.03	6.756	0.93	71.47
	100	1.41	3.4	0.83	71.18
	150	1.90	3.17	0.89	81.78
	180	1.68	26.96	0.93	76.17

*In C/N, pH and shaking speed, glucose was chosen as carbon source.*

Previous results have shown that strain Y-9 performs DNRA and nitrate assimilation under aerobic conditions (Huang et al., 2020). Based on the duration of cultivation (4 d), the detectable NH_4_^+^ in the supernatant might have resulted from DNRA and nitrate assimilation followed by mineralization (Figure 1C). It is worth noting that after 3 d of cultivation, when all the cells were in the stationary phase, the detectable NH_4_^+^ began to decrease in the glucose-containing system. However, it continued to increase in the media containing sodium acetate and sodium citrate. These results demonstrated that NO_3_^–^ reduction by strain Y-9 differed when glucose was the sole carbon source compared to the other two carbon sources.

**FIGURE 2:**
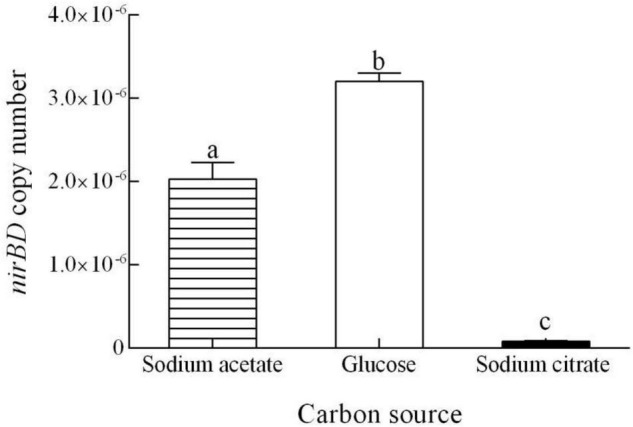
Quantitative measurement of the *nirBD* expression in *Pseudomonas putida* Y-9 cultured with different carbon sources for 4 d. The values are expressed as the number of copies/10^10^ copies of 16S rRNA. The different lowercase letters above the bars indicate significant differences among treatments (*P* < 0.05).

The TN decreases in our system were due to the denitrification activities of strain Y-9, and *nirBD* in strain Y-9 controls DNRA and nitrate assimilation (Huang et al., 2020). The TN in the media supplemented with different carbon sources tended to decrease (Figure 1D). The maximum TN decrease (60.27 mg/L) was found in the sodium citrate-containing medium, and the minimum TN decrease occurred (22.77 mg/L) in the glucose medium. This was in accordance with data from Yang et al. (2012) who reported that *Pseudomonas stutzeri* D6 most effectively removed TN when sodium citrate was the carbon source. Moreover, the *nirBD* expression level in strain Y-9 peaked when glucose was the carbon source (Figure 2). These findings demonstrated that glucose addition promoted DNRA and nitrate assimilation, effectively removing most of the NO_3_^–^ from the system (up to 89.79%) while inhibiting denitrification (i.e., the total nitrogen lost from the system was 22.77 mg/L).

### Effects of the C/N Ratio on Nitrate Reduction

**FIGURE 3:**
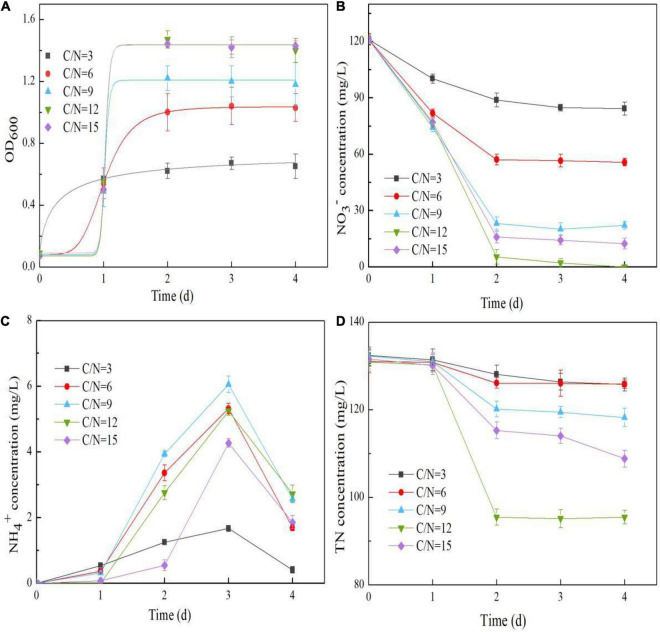
Effects of the C/N ratio on the OD_600_ **(A)**, NO_3_^–^ concentration **(B)**, NH_4_^+^ concentration **(C)**, and TN concentration **(D)** in the *Pseudomonas putida* Y-9 culture with glucose as the sole carbon source.

The effects of the C/N ratio on the nitrate reduction conducted by strain Y-9 were further studied. Strain Y-9 growth improved as the C/N ratio increased (Figure 3A). This result was consistent with previous studies (Kim et al., 2008; Liu et al., 2016), that the growth of *P. putida* AD-21 and *Marinobacter* strain NNA5 increased as the relative proportion of carbon increased in the medium. This might have been because electron transfer slowed when carbon concentrations were low, providing insufficient energy for microbial growth (Kim et al., 2008; Zhao et al., 2018). Greater than 80% of the NO_3_^–^ was removed at C/N ratios of 9–15. However, the removal efficiency of NO_3_^–^ did not exceed 30.46 and 54.00% when the C/N ratio was 3 and 6, respectively (Figure 3B). Furthermore, nitrate degradation rates at C/N ratios of 9–15 were consistent with the predictions of the modified Compertz model (*R*^2^ > 0.90), and the NO_3_^–^ conversion rate was maximum at a C/N ratio of 12 (Table 1).

**FIGURE 4:**
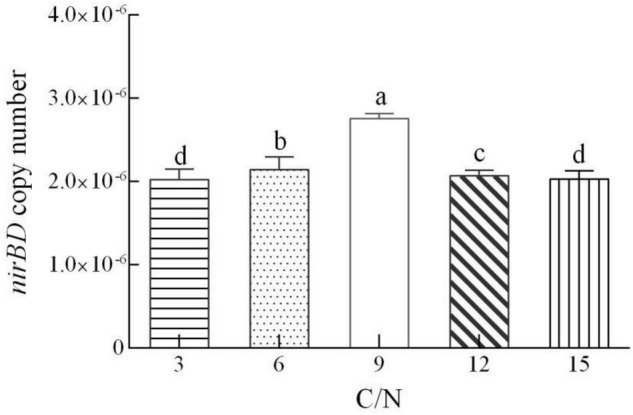
Quantitative measurement of the *nirBD* expression in *Pseudomonas putida* Y-9 cultured with different C/N ratios (with glucose as the sole carbon source) for 4 d. The values are expressed as the number of copies/10^10^ copies of 16S rRNA. The different lowercase letters above the bars indicate significant differences among treatments (*P* < 0.05).

The decrease of TN in this system generally mirrored the change in NO_3_^–^ (Figure 3D). The reduction in TN at the extremely high C/N ratio of 15 was lower than the reduction in TN at a C/N ratio of 12, suggesting that a C/N ratio of 12 was optimal for denitrification. Our results indicated that the influences of C/N ratios on Y-9-driven denitrification agreed with many previous studies. They showed that extremely low or high carbon concentrations suppressed microorganismal denitrification (Kim et al., 2008; Guo et al., 2016; Zhao et al., 2018). NH_4_^+^ concentration in the supernatant initially increased and then decreased during NO_3_^–^ reduction (Figure 3C), consistent with our carbon source analysis (Figure 1C). When the C/N ratio was 9, strain Y-9 removed most of the NO_3_^–^ (removal efficiency 81.78%) via DNRA and NO_3_^–^ assimilation (*nirBD* in strain Y-9 was most strongly expressed (Figure 4)). Notably, the denitrification performance of strain Y-9 at a C/N ratio of 9 was significantly weaker than that at a C/N ratio of 12 (*P* < 0.05).

### Effects of the Initial pH on Nitrate Reduction

**FIGURE 5:**
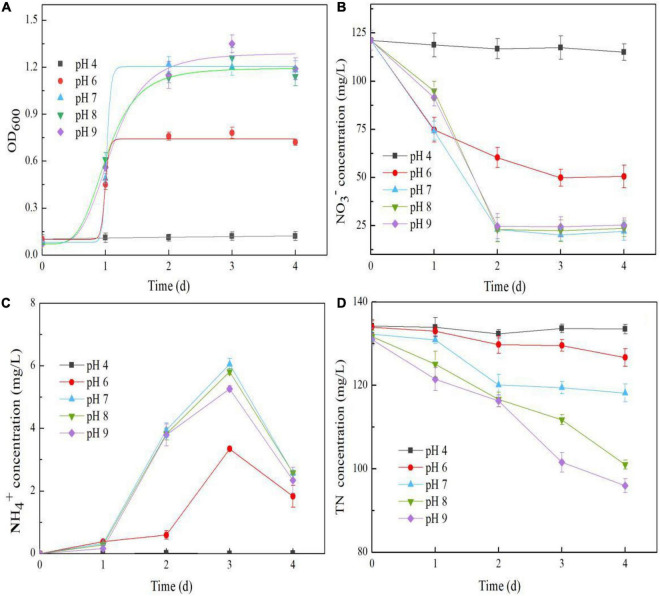
Effects of pH on the OD_600_ **(A)**, NO_3_^–^ concentration **(B)**, NH_4_^+^ concentration **(C)**, and TN concentration **(D)** in the *Pseudomonas putida* Y-9 culture with glucose as the carbon source.

The impacts of the initial pH on the nitrate reduction performance of strain Y-9 are shown in Figure 5. At an initial pH of 4, the bacterial density did not noticeably increase, and NO_3_^–^ reduction was minimal throughout the experiment (Figures 5A,B), suggesting that an overly acidic environment was detrimental to these bacteria. However, in the pH range 7–9, strain growth and NO_3_^–^ removal were significantly improved (*P* < 0.05) (Figures 5A,B). These results are in agreement with the general finding that neutral or alkaline environments are beneficial for bacteria growth and bacterium-driven NO_3_^–^ removal (Li et al., 2017; Rout et al., 2017). The NO_3_^–^ removal efficiency was significantly positively correlated with the growth of strain Y-9 (*P* < 0.01) (Figure 5), indicating that pH might control the NO_3_^–^ removal efficiency by influencing the growth of strain Y-9. However, this possibility requires further study. The TN concentration in the suspension decreased as the initial pH increased, and the TN decreased by 35.01 mg/L at pH 9 (Figure 5D). This indicated that alkaline environments favored the denitrification in strain Y-9 under aerobic conditions.

**FIGURE 6:**
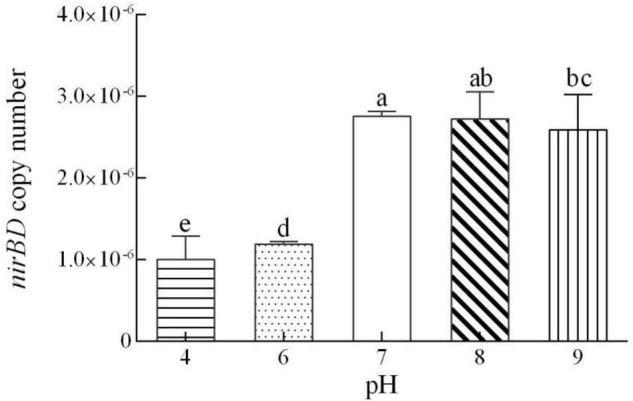
Quantitative measurement of the *nirBD* expression in *Pseudomonas putida* Y-9 cultured in different initial pH mediums (with glucose as the sole carbon source) for 4 d. The values are expressed as the number of copies/10^10^ copies of 16S rRNA. The different lowercase letters above the bars indicate significant differences among treatments (*P* < 0.05).

After 3 d of culture, a negligible amount of NH_4_^+^ was detected at pH 4. However, the accumulation of NH_4_^+^ at pH 7–9 was higher than 5.0 mg/L (Figure 5C). At the end of the experiment, the *nirBD* expression level in strain Y-9 at pH 7–9 was better than at pH 4 or 6 (Figure 6). These results showed that the initial pH affected the expression of *nirBD* in strain Y-9, and this might influence NH_4_^+^ production from DNRA and NO_3_^–^ assimilation as well as its subsequent mineralization (Huang et al., 2020). The results of previous studies on the effects of pH on NO_3_^–^ reduction by soil microorganisms are widely contradictory (Nägele and Conrad, 1990; Stevens et al., 1998). Here, strain Y-9 effectively performed NO_3_^–^ assimilation, DNRA, and denitrification at pH 7–9. This finding was inconsistent with a previous study (Yoon et al., 2015) that suggested that a low pH was more favorable for denitrification, while a high pH promoted the production of NH_4_^+^ via DNRA. This discrepancy indicated that the effects of pH on the microbial nitrogen cycle were complex and required further study. Our results suggested that a neutral pH was most favorable for NO_3_^–^ removal and nitrogen retention.

### Effects of Dissolved Oxygen on Nitrate Reduction

**FIGURE 7:**
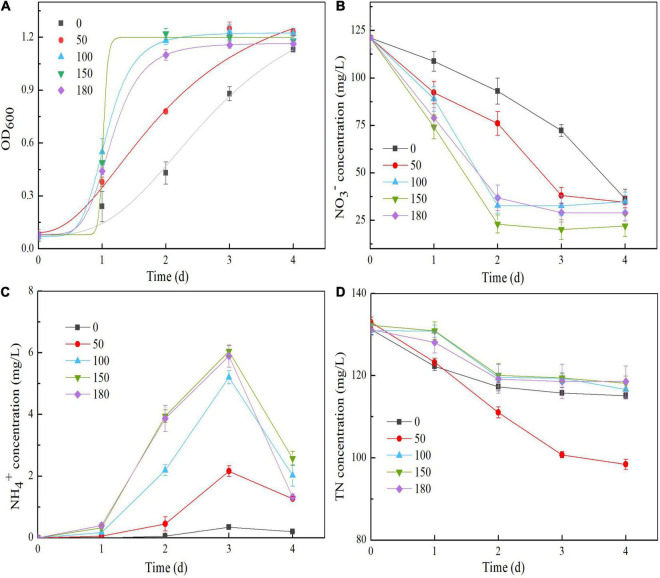
Effects of shaking speed on the OD_600_ **(A)**, NO_3_^–^ concentration **(B)**, NH_4_^+^ concentration **(C)**, and TN concentration **(D)** in the *Pseudomonas putida* Y-9 culture with glucose as the sole carbon source.

Strain Y-9 growth and NO_3_^–^ reduction increased gradually as the shaking speed increased (Figures 7A,B). Changes in the NO_3_^–^ degradation rates at various rotating speeds were consistent with the predictions of the modified Compertz model (*R*^2^ > 0.80), and the NO_3_^–^ conversion rate achieved its maximum at 150 rpm (Table 1). These results suggested that increasingly aerobic conditions improved strain growth and NO_3_^–^ reduction. TN decreased gradually throughout the incubation process, irrespective of the DO concentration. However, TN decreased at low shaking speeds (≤50 rpm) was significantly greater than those at high shaking speeds (≥100 rpm) (*P* < 0.05). The decrease in the TN at the end of the experiment reached the maximum (34.58 mg/L) at a shaking speed of 50 rpm (Figure 7D). These results suggested that the denitrification performance of strain Y-9 first increased and then decreased as the DO concentration increased. This finding was consistent with the results of Zhao et al. (2018); Rout et al. (2017), and Huang and Tseng (2001). Previous studies demonstrated that the denitrification performance remained stable as long as the DO concentration remained within a fixed range. Nevertheless, the denitrification enzyme activity levels improved noticeably when the DO concentration decreased below a threshold value (Song et al., 2011). For strain Y-9, 50 rpm might be the threshold DO value that affects denitrification enzymes, although this possibility requires further testing.

**FIGURE 8:**
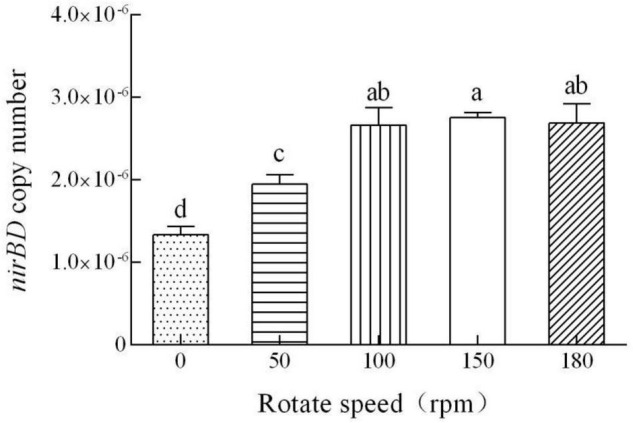
Quantitative measurement of the *nirBD* expression in *Pseudomonas putida* Y-9 cultured at different shaking speeds (with glucose as the sole carbon source) for 4 d. The values are expressed as the number of copies/10^10^ copies of 16S rRNA. The different lowercase letters above the bars indicate significant differences among treatments (*P* < 0.05).

The quantitative PCR amplification results indicated that the expression of *nirBD* increased with an increase in the shaking speed (Figure 8). These results, in conjunction with the NO_3_^–^ reduction performance of strain Y-9 (Huang et al., 2020), indicated that high DO concentrations stimulated the expression of *nirBD* in strain Y-9, promoting NO_3_^–^ assimilation as well as DNRA, and thus releasing more NH_4_^+^ into the supernatant (Figure 7C). Consistent with this, Yang et al. (2012) and Zhao et al. (2018) found that NH_4_^+^ production increased with the DO content during NO_3_^–^ reduction by *P. stutzeri* D6 and *P. stutzeri* strain XL-2. Variance analyses indicated that the amounts of NO_3_^–^ removal from the culture media and NH_4_^+^ accumulated in the culture media at high shaking speeds (≥100 rpm) differed obviously from those at low rotation speeds (≤50 rpm) (*P* < 0.05). These results indicated that good aeration effectively promoted NO_3_^–^ removal and NH_4_^+^ production by strain Y-9.

The heavy application of chemical nitrogen fertilizers leads to an accumulation of highly mobile nitrate in upland soils and significantly increases the risk of nitrogen loss (Lin et al., 2020; Vidal et al., 2020). Therefore, it is essential to control NO_3_^–^ concentrations in soil. The nitrogen cycle conducted by microorganisms plays a critical role in regulating nitrate concentrations in soil, compared to artificially limiting the application of ammonium and nitrate fertilizers (Shao et al., 2011; Song et al., 2014; Zhang et al., 2015; Pandey et al., 2019; Wang et al., 2020). Denitrification effectively removes excess NO_3_^–^ from soil systems but leads to nitrogen losses in the form of nitrogen gas or the greenhouse gas N_2_O (Stein and Klotz, 2016). For example, Putz et al. (2018) showed that approximately 70–78% of all N_2_O originated from denitrification in annual cereal soils. Both the DNRA and NO_3_^–^ assimilation processes can decrease soil NO_3_^–^ concentration and facilitate soil nitrogen conservation by reducing NO_3_^–^ to NH_4_^+^ via NO_2_^–^ (Kuypers et al., 2018; Wang et al., 2020). Thus, to pursue the minimum loss of nitrogen and maximize nitrogen fertilizer efficiency, strategies that strengthen DNRA as well as NO_3_^–^ assimilation while weakening denitrification in surface soils should be pursued. Previously, we found that strain Y-9 performs simultaneous nitrate assimilation, DNRA, and denitrification under aerobic conditions. It has also been clarified that the gene, *nirBD*, controls NO_3_^–^ assimilation and DNRA process in strain Y-9 (Huang et al., 2020). In this study, we further explored the environmental factors that affect the nitrate removal pathways of strain Y-9. Our results provide a theoretical reference for technical studies of nitrate removal and nitrogen conservation in farmland soils.

## Conclusion

Four common external environmental conditions (carbon source, C/N ratio, pH, and dissolved oxygen) affected the nitrate reduction performance of strain Y-9. A high initial pH enhanced nitrate assimilation, denitrification, and the DNRA of strain Y-9.

The optimal conditions for the nitrate assimilation and the DNRA of strain Y-9 were glucose as the carbon source, C/N 9, pH 7.0, and 150 rpm. Under these conditions, the nitrogen loss from the system was the smallest.

## Data Availability Statement

The original contributions presented in the study are included in the article/Supplementary Material, further inquiries can be directed to the corresponding authors.

## Author Contributions

XH and ZL: conceptualization. XH: methodology, data curation, visualization, supervision, and writing—original draft preparation. WT: software, formal analysis, and investigation. XH, ZL, and DX: validation. ZL: resources and project administration. XH, ZL, DJ, and DX: writing—review and editing. XH and ZL: funding acquisition. All authors have read and agreed to the published version of the manuscript.

## Conflict of Interest

The authors declare that the research was conducted in the absence of any commercial or financial relationships that could be construed as a potential conflict of interest.

## Publisher’s Note

All claims expressed in this article are solely those of the authors and do not necessarily represent those of their affiliated organizations, or those of the publisher, the editors and the reviewers. Any product that may be evaluated in this article, or claim that may be made by its manufacturer, is not guaranteed or endorsed by the publisher.
