# Supplementary material for: Certain Environmental Conditions Maximize Ammonium Accumulation and Minimize Nitrogen Loss During Nitrate Reduction Process by Pseudomonas putida Y-9
Source: Front Microbiol. 2021 Dec 13;12:764241. doi: 10.3389/fmicb.2021.764241 (PMC8710668; doi:10.3389/fmicb.2021.764241)

Nonlinear Curve Fit (Compertz2 (User)) (2020/6/21 12:38:16)

Parameters

		Value	Standard Error
C	a	4.92557	1.10033
	b	23.99182	2.6365

Reduced Chi-sqr = 4.09213580611
COD(R^2) = 0.97989632370109
Iterations Performed = 8
Total Iterations in Session = 8
Fit converged. Chi-Sqr tolerance value of 1E-9 was reached.

Statistics

	C
Number of Points	5
Degrees of Freedom	3
Reduced Chi-Sqr	4.09214
Residual Sum of Squares	12.27641
Adj. R-Square	0.9732
Fit Status	Succeeded(100)

Fit Status Code :
100 : Fit converged. Chi-Sqr tolerance value of 1E-9 was reached.

Summary

	a		b		Statistics	
	Value	Standard Error	Value	Standard Error	Reduced Chi-Sqr	Adj. R-Square
C	4.92557	1.10033	23.99182	2.6365	4.09214	0.9732

ANOVA

		DF	Sum of Squares	Mean Square	F Value	Prob>F
C	Regression	2	1819.25347	909.62673	222.28655	6.54805E-4
	Residual	3	12.27641	4.09214		
	Uncorrected Total	5	1831.52987			
	Corrected Total	4	610.65485			

Fitted Curves Plot

C

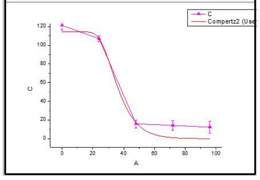

Residual vs. Independent Plot

C

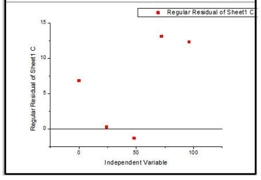

Nonlinear Curve Fit (Compertz2 (User)) (2020/6/21 12:37:47)

Parameters

		Value	Standard Error
B	a	1.6003	0.40492
	b	20.97222	9.00934

Reduced Chi-sqr = 3.7845435857
COD(R^2) = 0.95301446972127
Iterations Performed = 14
Total Iterations in Session = 14
Fit converged. Chi-Sqr tolerance value of 1E-9 was reached.

Statistics

	B
Number of Points	5
Degrees of Freedom	3
Reduced Chi-Sqr	3.78454
Residual Sum of Squares	11.35363
Adj. R-Square	0.93735
Fit Status	Succeeded(100)

Fit Status Code :
100 : Fit converged. Chi-Sqr tolerance value of 1E-9 was reached.

Summary

	a		b		Statistics	
	Value	Standard Error	Value	Standard Error	Reduced Chi-Sqr	Adj. R-Square
B	1.6003	0.40492	20.97222	9.00934	3.78454	0.93735

ANOVA

		DF	Sum of Squares	Mean Square	F Value	Prob>F
B	Regression	2	1198.40282	599.20141	158.32858	0.00108
	Residual	3	11.35363	3.78454		
	Uncorrected Total	5	1209.75645			
	Corrected Total	4	241.64101			

Fitted Curves Plot

B

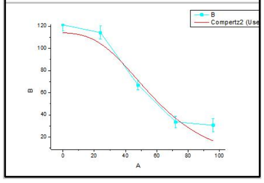

Residual vs. Independent Plot

B

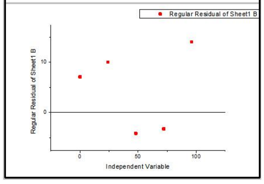

Nonlinear Curve Fit (Compertz2 (User)) (2021/9/7 11:47:59)

Parameters

		Value	Standard Error
D	a	44.35214	1.87668E8
	b	23.81456	852571.21252

Reduced Chi-sqr = 15.6408333334
COD(R^2) = 0.99690039325861
Iterations Performed = 33
Total Iterations in Session = 33
Fit did not converge - mutual dependency exists between parameters.
You may have overparameterized the fitting function. Fixing one of them may eliminate this problem.

Statistics

	D
Number of Points	5
Degrees of Freedom	3
Reduced Chi-Sqr	15.64083
Residual Sum of Squares	46.9225
Adj. R-Square	0.99587
Fit Status	Failed(-203)

Fit Status Code :
-203 : Fit did not converge - mutual dependency exists between parameters.
You may have overparameterized the fitting function. Fixing one of them may eliminate this problem

Summary

	a		b		Statistics	
	Value	Standard Error	Value	Standard Error	Reduced Chi-Sqr	Adj. R-Square
D	44.35214	1.87668E8	23.81456	852571.21252	15.64083	0.99587

ANOVA

		DF	Sum of Squares	Mean Square	F Value	Prob>F
D	Regression	2	25062.0799	12531.03995	801.1747	9.68126E-5
	Residual	3	46.9225	15.64083		
	Uncorrected Total	5	25109.0024			
	Corrected Total	4	15138.21072			

Fitted Curves Plot

D

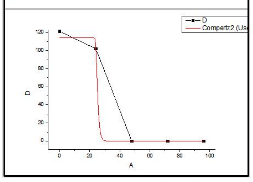

Residual vs. Independent Plot

D

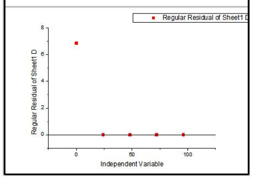

Supplement: Supplementary file 1 [file Data_Sheet_1.PDF]
