# Supplementary material for: Certain Environmental Conditions Maximize Ammonium Accumulation and Minimize Nitrogen Loss During Nitrate Reduction Process by Pseudomonas putida Y-9
Source: Front Microbiol. 2021 Dec 13;12:764241. doi: 10.3389/fmicb.2021.764241 (PMC8710668; doi:10.3389/fmicb.2021.764241)

Nonlinear Curve Fit (Compertz2 (User)) (2020/6/21 12:46:09)

Parameters

		Value	Standard Error
C	a	0.57576	0.23737
	b	-21.10828	33.36914

Reduced Chi-sqr = 40.8797563005
COD(R^2) = 0.7285799004276
Iterations Performed = 9
Total Iterations in Session = 9
Fit converged. Chi-Sqr tolerance value of 1E-9 was reached.

Statistics

	C
Number of Points	5
Degrees of Freedom	3
Reduced Chi-Sqr	40.87976
Residual Sum of Squares	122.63927
Adj. R-Square	0.63811
Fit Status	Succeeded(100)

Fit Status Code :
100 : Fit converged. Chi-Sqr tolerance value of 1E-9 was reached.

Summary

	a		b		Statistics	
	Value	Standard Error	Value	Standard Error	Reduced Chi-Sqr	Adj. R-Square
C	0.57576	0.23737	-21.10828	33.36914	40.87976	0.63811

ANOVA

		DF	Sum of Squares	Mean Square	F Value	Prob>F
C	Regression	2	4789.76017	2394.88008	58.58352	0.00463
	Residual	3	122.63927	40.87976		
	Uncorrected Total	5	4912.39944			
	Corrected Total	4	451.84299			

Fitted Curves Plot

C

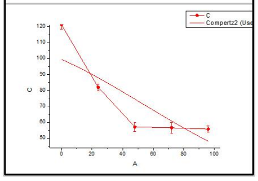

Residual vs. Independent Plot

C

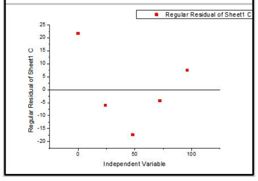

Nonlinear Curve Fit (Compertz2 (User)) (2020/6/21 12:47:05)

Parameters

		Value	Standard Error
D	a	1.91339	0.77181
	b	5.16867	10.86143

Reduced Chi-sqr = 44.0751017181
COD(R^2) = 0.92760881230048
Iterations Performed = 9
Total Iterations in Session = 9
Fit converged. Chi-Sqr tolerance value of 1E-9 was reached.

Statistics

	D
Number of Points	5
Degrees of Freedom	3
Reduced Chi-Sqr	44.0751
Residual Sum of Squares	132.22531
Adj. R-Square	0.90348
Fit Status	Succeeded(100)

Fit Status Code :
100 : Fit converged. Chi-Sqr tolerance value of 1E-9 was reached.

Summary

	a		b		Statistics	
	Value	Standard Error	Value	Standard Error	Reduced Chi-Sqr	Adj. R-Square
D	1.91339	0.77181	5.16867	10.86143	44.0751	0.90348

ANOVA

		DF	Sum of Squares	Mean Square	F Value	Prob>F
D	Regression	2	6061.6139	3030.80695	68.7646	0.00367
	Residual	3	132.22531	44.0751		
	Uncorrected Total	5	6193.8392			
	Corrected Total	4	1826.53869			

Fitted Curves Plot

D

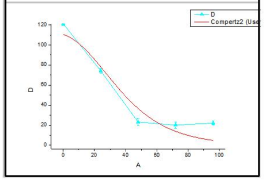

Residual vs. Independent Plot

D

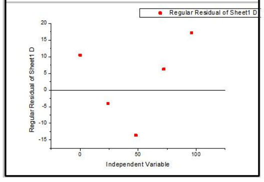

Nonlinear Curve Fit (Compertz2 (User)) (2020/6/21 12:44:39)

Parameters

		Value	Standard Error
G	a	0.43934	0.18062
	b	9.3549	24.82989

Reduced Chi-sqr = 9.58324528716
COD(R^2) = 0.77058684946571
Iterations Performed = 9
Total Iterations in Session = 9
Fit converged. Chi-Sqr tolerance value of 1E-9 was reached.

Statistics

	G
Number of Points	5
Degrees of Freedom	3
Reduced Chi-Sqr	9.58325
Residual Sum of Squares	28.74974
Adj. R-Square	0.69412
Fit Status	Succeeded(100)

Fit Status Code :
100 : Fit converged. Chi-Sqr tolerance value of 1E-9 was reached.

Summary

	a		b		Statistics	
	Value	Standard Error	Value	Standard Error	Reduced Chi-Sqr	Adj. R-Square
G	0.43934	0.18062	9.3549	24.82989	9.58325	0.69412

ANOVA

		DF	Sum of Squares	Mean Square	F Value	Prob>F
G	Regression	2	6775.48015	3387.74008	353.50656	3.2845E-4
	Residual	3	28.74974	9.58325		
	Uncorrected Total	5	6804.22989			
	Corrected Total	4	125.3186			

Fitted Curves Plot

G

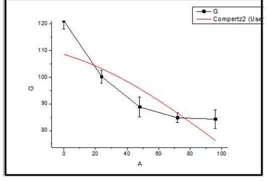

Residual vs. Independent Plot

G

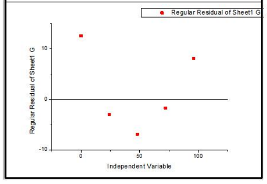

Nonlinear Curve Fit (Compertz2 (User)) (2020/6/21 12:48:01)

Parameters

		Value	Standard Error
F	a	2.99911	1.21978
	b	11.68154	6.60066

Reduced Chi-sqr = 21.8978434825
COD(R^2) = 0.97567169080392
Iterations Performed = 14
Total Iterations in Session = 14
Fit converged. Chi-Sqr tolerance value of 1E-9 was reached.

Statistics

	F
Number of Points	5
Degrees of Freedom	3
Reduced Chi-Sqr	21.89784
Residual Sum of Squares	65.69353
Adj. R-Square	0.96756
Fit Status	Succeeded(100)

Fit Status Code :
100 : Fit converged. Chi-Sqr tolerance value of 1E-9 was reached.

Summary

	a		b		Statistics	
	Value	Standard Error	Value	Standard Error	Reduced Chi-Sqr	Adj. R-Square
F	2.99911	1.21978	11.68154	6.60066	21.89784	0.96756

ANOVA

		DF	Sum of Squares	Mean Square	F Value	Prob>F
F	Regression	2	10443.00082	5221.50041	238.44816	5.90013E-4
	Residual	3	65.69353	21.89784		
	Uncorrected Total	5	10508.69435			
	Corrected Total	4	2700.29166			

Fitted Curves Plot

F

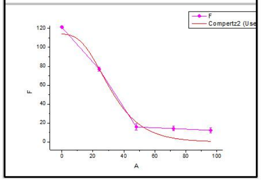

Residual vs. Independent Plot

F

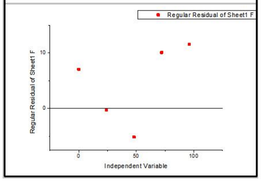

Nonlinear Curve Fit (Compertz2 (User)) (2020/6/21 12:50:01)

Parameters

		Value	Standard Error
E	a	5.44044	1.36599
	b	16.99485	2.00252

Reduced Chi-sqr = 16.790166204
COD(R^2) = 0.99584944606764
Iterations Performed = 8
Total Iterations in Session = 8
Fit converged. Chi-Sqr tolerance value of 1E-9 was reached.

Statistics

	E
Number of Points	5
Degrees of Freedom	3
Reduced Chi-Sqr	16.79017
Residual Sum of Squares	50.3705
Adj. R-Square	0.99447
Fit Status	Succeeded(100)

Fit Status Code :
100 : Fit converged. Chi-Sqr tolerance value of 1E-9 was reached.

Summary

	a		b		Statistics	
	Value	Standard Error	Value	Standard Error	Reduced Chi-Sqr	Adj. R-Square
E	5.44044	1.36599	16.99485	2.00252	16.79017	0.99447

ANOVA

		DF	Sum of Squares	Mean Square	F Value	Prob>F
E	Regression	2	20473.268	10236.634	609.68033	1.45633E-4
	Residual	3	50.3705	16.79017		
	Uncorrected Total	5	20523.6385			
	Corrected Total	4	12135.84968			

Fitted Curves Plot

E

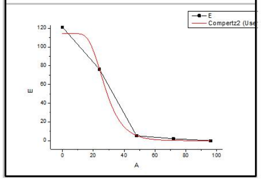

Residual vs. Independent Plot

E

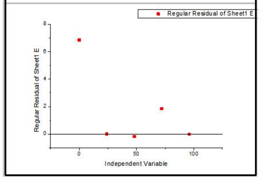

Supplement: Supplementary file 2 [file Data_Sheet_2.PDF]
