# Supplementary material for: Certain Environmental Conditions Maximize Ammonium Accumulation and Minimize Nitrogen Loss During Nitrate Reduction Process by Pseudomonas putida Y-9
Source: Front Microbiol. 2021 Dec 13;12:764241. doi: 10.3389/fmicb.2021.764241 (PMC8710668; doi:10.3389/fmicb.2021.764241)

Nonlinear Curve Fit (Compertz2 (User)) (2020/6/21 12:57:11)

Parameters

		Value	Standard Error
B	a	-1.62929	--
	b	-3177.12844	--

Reduced Chi-sqr = 0.942880955666
Iterations Performed = 0
Total Iterations in Session = 0
Fit did not converge - reason unknown.
Fitting was done with weights. Check weighting method and error bar values for possible inconsistencies, or try fitting again with weights turned off.

Statistics

	B
Number of Points	5
Degrees of Freedom	3
Reduced Chi-Sqr	0.94288
Residual Sum of Squares	2.82864
Adj. R-Square	-3.20696
Fit Status	Failed(-207)

Fit Status Code :
-207 : Fit did not converge - reason unknown.
Fitting was done with weights. Check weighting method and error bar values for possible inconsistencies, or try fitting again with weights turned o

Summary

	a		b		Statistics	
	Value	Standard Error	Value	Standard Error	Reduced Chi-Sqr	Adj. R-Square
B	-1.62929	--	-3177.12844	--	0.94288	-3.20696

ANOVA

		DF	Sum of Squares	Mean Square	F Value	Prob>F
B	Regression	2	2546.89325	1273.44662	1350.5911	4.43127E-5
	Residual	3	2.82864	0.94288		
	Uncorrected Total	5	2549.72189			
	Corrected Total	4	0.8965			

Fitted Curves Plot

B

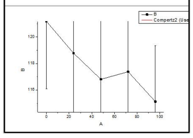

Residual vs. Independent Plot

B

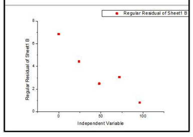

Nonlinear Curve Fit (Compertz2 (User)) (2020/6/21 12:53:09)

Parameters

		Value	Standard Error
G	a	0.80029	0.29712
	b	-7.73337	24.41946

Reduced Chi-sqr = 8.75344871151
COD(R^2) = 0.80759053425098
Iterations Performed = 8
Total Iterations in Session = 8
Fit converged - Chi-sqr no longer changed.

Statistics

	G
Number of Points	5
Degrees of Freedom	3
Reduced Chi-Sqr	8.75345
Residual Sum of Squares	26.26035
Adj. R-Square	0.74345
Fit Status	Succeeded(101)

Fit Status Code :
101 : Fit converged - Chi-sqr no longer changed.

Summary

	a		b		Statistics	
	Value	Standard Error	Value	Standard Error	Reduced Chi-Sqr	Adj. R-Square
G	0.80029	0.29712	-7.73337	24.41946	8.75345	0.74345

ANOVA

		DF	Sum of Squares	Mean Square	F Value	Prob>F
G	Regression	2	1011.27103	505.63552	57.76415	0.00473
	Residual	3	26.26035	8.75345		
	Uncorrected Total	5	1037.53138			
	Corrected Total	4	136.48157			

Fitted Curves Plot

G

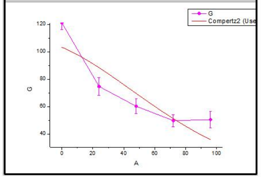

Residual vs. Independent Plot

G

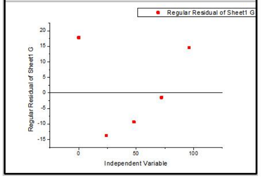

Nonlinear Curve Fit (Compertz2 (User)) (2021/9/7 12:16:20)

Parameters

		Value	Standard Error
C	a	2.04608	0.72792
	b	4.87285	10.92977

Reduced Chi-sqr = 206.10132755
COD(R^2) = 0.92295317030284
Iterations Performed = 7
Total Iterations in Session = 7
Fit converged. Chi-Sqr tolerance value of 1E-9 was reached.

Statistics

	C
Number of Points	5
Degrees of Freedom	3
Reduced Chi-Sqr	206.10133
Residual Sum of Squares	618.30398
Adj. R-Square	0.89727
Fit Status	Succeeded(100)

Fit Status Code :
100 : Fit converged. Chi-Sqr tolerance value of 1E-9 was reached

Summary

	a		b		Statistics	
	Value	Standard Error	Value	Standard Error	Reduced Chi-Sqr	Adj. R-Square
C	2.04608	0.72792	4.87285	10.92977	206.10133	0.89727

ANOVA

		DF	Sum of Squares	Mean Square	F Value	Prob>F
C	Regression	2	21004.84972	10502.42486	50.95758	0.00566
	Residual	3	618.30398	206.10133		
	Uncorrected Total	5	21623.1537			
	Corrected Total	4	8025.0412			

Fitted Curves Plot

C

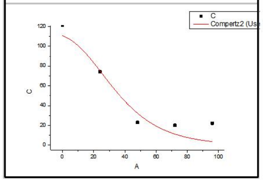

Residual vs. Independent Plot

C

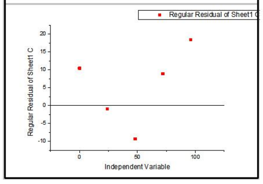

Nonlinear Curve Fit (Compertz2 (User)) (2020/6/21 12:55:20)

Parameters

		Value	Standard Error
D	a	1.91347	0.77578
	b	10.98678	12.4096

Reduced Chi-sqr = 10.963810862
COD(R^2) = 0.90425445552089
Iterations Performed = 18
Total Iterations in Session = 18
Fit converged. Chi-Sqr tolerance value of 1E-9 was reached.

Statistics

	D
Number of Points	5
Degrees of Freedom	3
Reduced Chi-Sqr	10.96381
Residual Sum of Squares	32.89143
Adj. R-Square	0.87234
Fit Status	Succeeded(100)

Fit Status Code :
100 : Fit converged. Chi-Sqr tolerance value of 1E-9 was reached.

Summary

	a		b		Statistics	
	Value	Standard Error	Value	Standard Error	Reduced Chi-Sqr	Adj. R-Square
D	1.91347	0.77578	10.98678	12.4096	10.96381	0.87234

ANOVA

		DF	Sum of Squares	Mean Square	F Value	Prob>F
D	Regression	2	967.07106	483.53553	44.10287	0.00696
	Residual	3	32.89143	10.96381		
	Uncorrected Total	5	999.9625			
	Corrected Total	4	343.52964			

Fitted Curves Plot

D

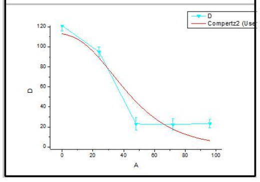

Residual vs. Independent Plot

D

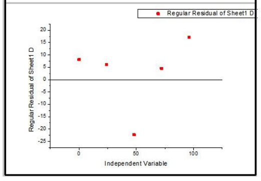

Nonlinear Curve Fit (Compertz2 (User)) (2020/6/21 12:56:11)

Parameters

		Value	Standard Error
H	a	1.49547	0.50659
	b	6.0383	13.61767

Reduced Chi-sqr = 12.1165574394
COD(R^2) = 0.89987236759647
Iterations Performed = 14
Total Iterations in Session = 14
Fit converged. Chi-Sqr tolerance value of 1E-9 was reached.

Statistics

	H
Number of Points	5
Degrees of Freedom	3
Reduced Chi-Sqr	12.11656
Residual Sum of Squares	36.34967
Adj. R-Square	0.8665
Fit Status	Succeeded(100)

Fit Status Code :
100 : Fit converged. Chi-Sqr tolerance value of 1E-9 was reached.

Summary

	a		b		Statistics	
	Value	Standard Error	Value	Standard Error	Reduced Chi-Sqr	Adj. R-Square
H	1.49547	0.50659	6.0383	13.61767	12.11656	0.8665

ANOVA

		DF	Sum of Squares	Mean Square	F Value	Prob>F
H	Regression	2	1073.06775	536.53388	44.28105	0.00692
	Residual	3	36.34967	12.11656		
	Uncorrected Total	5	1109.41742			
	Corrected Total	4	363.03337			

Fitted Curves Plot

H

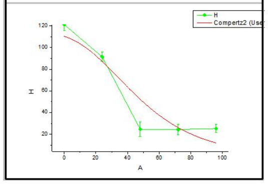

Residual vs. Independent Plot

H

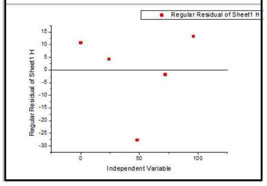

Supplement: Supplementary file 3 [file Data_Sheet_3.PDF]
