# Supplementary material for: Certain Environmental Conditions Maximize Ammonium Accumulation and Minimize Nitrogen Loss During Nitrate Reduction Process by Pseudomonas putida Y-9
Source: Front Microbiol. 2021 Dec 13;12:764241. doi: 10.3389/fmicb.2021.764241 (PMC8710668; doi:10.3389/fmicb.2021.764241)

Nonlinear Curve Fit (Compertz2 (User)) (2020/6/21 13:01:47)

Parameters

		Value	Standard Error
B	a	1.33405	0.25022
	b	38.6994	7.02381

Reduced Chi-sqr = 1.36743966844
COD(R^2) = 0.97522775475204
Iterations Performed = 12
Total Iterations in Session = 12
Fit converged. Chi-Sqr tolerance value of 1E-9 was reached.

Statistics

	B
Number of Points	5
Degrees of Freedom	3
Reduced Chi-Sqr	1.36744
Residual Sum of Squares	4.10232
Adj. R-Square	0.96697
Fit Status	Succeeded(100)

Fit Status Code :
100 : Fit converged. Chi-Sqr tolerance value of 1E-9 was reached.

Summary

	a		b		Statistics	
	Value	Standard Error	Value	Standard Error	Reduced Chi-Sqr	Adj. R-Square
B	1.33405	0.25022	38.6994	7.02381	1.36744	0.96697

ANOVA

		DF	Sum of Squares	Mean Square	F Value	Prob>F
B	Regression	2	1614.09904	807.04952	590.19022	1.52876E-4
	Residual	3	4.10232	1.36744		
	Uncorrected Total	5	1618.20136			
	Corrected Total	4	165.60142			

Fitted Curves Plot

B

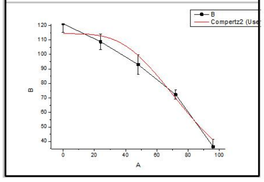

Residual vs. Independent Plot

B

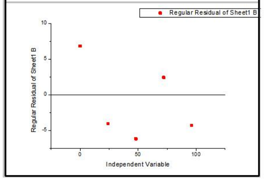

Nonlinear Curve Fit (Compertz2 (User)) (2020/6/21 13:02:42)

Parameters

		Value	Standard Error
G	a	1.03392	0.2093
	b	6.75578	13.14525

Reduced Chi-sqr = 4.16422646434
COD(R^2) = 0.94695904385606
Iterations Performed = 9
Total Iterations in Session = 9
Fit converged. Chi-Sqr tolerance value of 1E-9 was reached.

Statistics

	G
Number of Points	5
Degrees of Freedom	3
Reduced Chi-Sqr	4.16423
Residual Sum of Squares	12.49268
Adj. R-Square	0.92928
Fit Status	Succeeded(100)

Fit Status Code :
100 : Fit converged. Chi-Sqr tolerance value of 1E-9 was reached.

Summary

	a		b		Statistics	
	Value	Standard Error	Value	Standard Error	Reduced Chi-Sqr	Adj. R-Square
G	1.03392	0.2093	6.75578	13.14525	4.16423	0.92928

ANOVA

		DF	Sum of Squares	Mean Square	F Value	Prob>F
G	Regression	2	1004.58028	502.29014	120.62028	0.00162
	Residual	3	12.49268	4.16423		
	Uncorrected Total	5	1017.07296			
	Corrected Total	4	235.52893			

Fitted Curves Plot

G

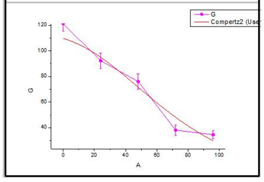

Residual vs. Independent Plot

G

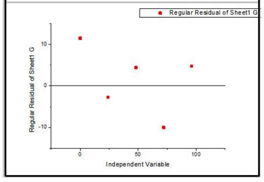

Nonlinear Curve Fit (Compertz2 (User)) (2020/6/21 13:06:07)

Parameters

		Value	Standard Error
B	a	1.41638	0.51761
	b	3.40409	15.66725

Reduced Chi-sqr = 294.746660515
COD(R^2) = 0.87183536418698
Iterations Performed = 12
Total Iterations in Session = 12
Fit converged. Chi-Sqr tolerance value of 1E-9 was reached.

Statistics

	B
Number of Points	5
Degrees of Freedom	3
Reduced Chi-Sqr	294.74666
Residual Sum of Squares	884.23998
Adj. R-Square	0.82911
Fit Status	Succeeded(100)

Fit Status Code :
100 : Fit converged. Chi-Sqr tolerance value of 1E-9 was reached.

Summary

	a		b		Statistics	
	Value	Standard Error	Value	Standard Error	Reduced Chi-Sqr	Adj. R-Square
B	1.41638	0.51761	3.40409	15.66725	294.74666	0.82911

ANOVA

		DF	Sum of Squares	Mean Square	F Value	Prob>F
B	Regression	2	24825.53542	12412.76771	42.11334	0.00743
	Residual	3	884.23998	294.74666		
	Uncorrected Total	5	25709.7754			
	Corrected Total	4	6899.25092			

Fitted Curves Plot

B

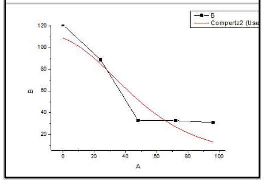

Residual vs. Independent Plot

B

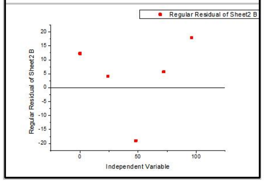

Nonlinear Curve Fit (Compertz2 (User)) (2020/6/21 13:07:12)

Parameters

		Value	Standard Error
D	a	1.89776	0.61603
	b	3.17158	12.08277

Reduced Chi-sqr = 6.88306153275
COD(R^2) = 0.91737852202579
Iterations Performed = 12
Total Iterations in Session = 12
Fit converged. Chi-Sqr tolerance value of 1E-9 was reached.

Statistics

	D
Number of Points	5
Degrees of Freedom	3
Reduced Chi-Sqr	6.88306
Residual Sum of Squares	20.64918
Adj. R-Square	0.88984
Fit Status	Succeeded(100)

Fit Status Code :
100 : Fit converged. Chi-Sqr tolerance value of 1E-9 was reached.

Summary

	a		b		Statistics	
	Value	Standard Error	Value	Standard Error	Reduced Chi-Sqr	Adj. R-Square
D	1.89776	0.61603	3.17158	12.08277	6.88306	0.88984

ANOVA

		DF	Sum of Squares	Mean Square	F Value	Prob>F
D	Regression	2	579.17435	289.58717	42.07244	0.00744
	Residual	3	20.64918	6.88306		
	Uncorrected Total	5	599.82353			
	Corrected Total	4	249.92514			

Fitted Curves Plot

D

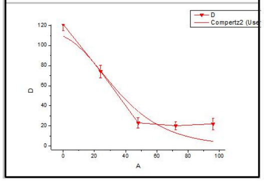

Residual vs. Independent Plot

D

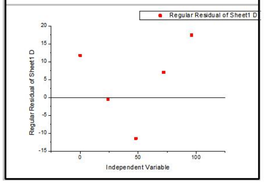

Supplement: Supplementary file 4 [file Data_Sheet_4.PDF]
